# Supplementary material for: Separate lanes for adding and reading in the white matter highways of the human brain
Source: Nat Commun. 2019 Aug 15;10:3675. doi: 10.1038/s41467-019-11424-1 (PMC6695422; doi:10.1038/s41467-019-11424-1)
Supplement: Supplementary file 3 — Reporting Summary [file 41467_2019_11424_MOESM3_ESM.pdf]

nature research

Corresponding author(s): Dr. Mareike Grotheer

Reporting Summary

Nature Research wishes to improve the reproducibility of the work that we publish. This form provides structure for consistency and transparency in reporting. For further information on Nature Research policies, see Authors & Referees and the Editorial Policy Checklist.

Statistical parameters

When statistical analyses are reported, confirm that the following items are present in the relevant location (e.g. figure legend, table legend, main text, or Methods section).

n/a	Confirmed	
☐	☒	The exact sample size (*n*) for each experimental group/condition, given as a discrete number and unit of measurement
☐	☒	An indication of whether measurements were taken from distinct samples or whether the same sample was measured repeatedly
☐	☒	The statistical test(s) used AND whether they are one- or two-sided<br>*Only common tests should be described solely by name; describe more complex techniques in the Methods section.*
☐	☒	A description of all covariates tested
☐	☒	A description of any assumptions or corrections, such as tests of normality and adjustment for multiple comparisons
☐	☒	A full description of the statistics including central tendency (e.g. means) or other basic estimates (e.g. regression coefficient) AND variation (e.g. standard deviation) or associated estimates of uncertainty (e.g. confidence intervals)
☐	☒	For null hypothesis testing, the test statistic (e.g. *F*, *t*, *r*) with confidence intervals, effect sizes, degrees of freedom and *P* value noted<br>*Give P values as exact values whenever suitable.*
☒	☐	For Bayesian analysis, information on the choice of priors and Markov chain Monte Carlo settings
☒	☐	For hierarchical and complex designs, identification of the appropriate level for tests and full reporting of outcomes
☒	☐	Estimates of effect sizes (e.g. Cohen's *d*, Pearson's *r*), indicating how they were calculated
☐	☒	Clearly defined error bars<br>*State explicitly what error bars represent (e.g. SD, SE, CI)*

Our web collection on statistics for biologists may be useful.

Software and code

Policy information about availability of computer code

Data collection	Code for stimulus display: PsychToolbox: http://psychtoolbox.org.<br>Custom code written in Matlab: Code will be made available upon request on our GitHub repository (https://github.com/VPNL/mrLanes).
Data analysis	Software packages used for data analyses:<br>Matlab version 2015a: https://www.mathworks.com/products/matlab.html<br>FreeSurfer version 5.3: https://surfer.nmr.mgh.harvard.edu<br>MRTrix3: http://www.mrtrix.org/download/<br>Automatic Fiber Quantification: https://github.com/yeatmanlab/AFQ<br>Vistasoft: https://github.com/vistalab/vistasoft<br>mrQ: https://github.com/mezera/mrQ<br><br>Custom code for dwi analysis:<br>https://github.com/vistalab/RTP-preproc<br>https://github.com/vistalab/RTP-pipeline<br>https://github.com/VPNL/mrLanes<br><br>Custom code for figures and statistics:

April 2018

https://github.com/VPNL/mrLanes

For manuscripts utilizing custom algorithms or software that are central to the research but not yet described in published literature, software must be made available to editors/reviewers upon request. We strongly encourage code deposition in a community repository (e.g. GitHub). See the Nature Research guidelines for submitting code & software for further information.

Data

Policy information about availability of data

All manuscripts must include a data availability statement. This statement should provide the following information, where applicable:

- Accession codes, unique identifiers, or web links for publicly available datasets
- A list of figures that have associated raw data
- A description of any restrictions on data availability

Data will be made available upon request. Source data for the figures as well as Matlab code used for figure generation is made available in our VPNL GitHub repository: https://github.com/VPNL/mrLanes

Field-specific reporting

Please select the best fit for your research. If you are not sure, read the appropriate sections before making your selection.

☒ Life sciences ☐ Behavioural & social sciences ☐ Ecological, evolutionary & environmental sciences

For a reference copy of the document with all sections, see nature.com/authors/policies/ReportingSummary-flat.pdf

Life sciences study design

All studies must disclose on these points even when the disclosure is negative.

Sample size	Sample size was chosen based on our previous study (Grotheer et al., 2018), which used the same functional MRI design as the current study, and in which we could successfully identify regions involved in math and reading in each individual participant's brain. We increased the sample size from the initial submission to the revised version from 14 to 20, in accordance with a reviewer's requests.
Data exclusions	None
Replication	N/A
Randomization	N/A; we only have a single set of participants
Blinding	N/A; we only have a single set of participants

Reporting for specific materials, systems and methods

Materials & experimental systems

n/a	Involved in the study
☒	☐ Unique biological materials
☒	☐ Antibodies
☒	☐ Eukaryotic cell lines
☒	☐ Palaeontology
☒	☐ Animals and other organisms
☐	☒ Human research participants

Methods

n/a	Involved in the study
☒	☐ ChIP-seq
☒	☐ Flow cytometry
☐	☒ MRI-based neuroimaging

Human research participants

Policy information about studies involving human research participants

Population characteristics	20 healthy adult volunteers (10 female, mean age ±SE: 27±1 years, 1 left-handed) participated in this study.
Recruitment	Participants were recruited from Stanford University and surrounding areas and participated in two experimental sessions.

Magnetic resonance imaging

Experimental design

Design type	Task based, mixed-design MRI
Design specifications	We used 7 s long trials. These were interleaved with gray blank screen, which served as a baseline. 48 trials were collected for each task.
Behavioral performance measures	We recorded button presses and response times during fMRI. Behavioral performance is included in the method section.

Acquisition

Imaging type(s)	functional MRI, diffusion MRI, anatomical MRI, quantitative MRI
Field strength	3T
Sequence & imaging parameters	Anatomical MRI: A whole-brain, anatomical volume was acquired, once for each participant, using a T1-weighted BRAVO pulse sequence (resolution: 1mm x 1 mm x 1 mm, TI=450 ms, flip angle: 12°, 1 NEX, FoV: 240 mm).<br><br>Functional MRI: We acquired 48 slices covering the occipitotemporal and most of the frontal cortex using a T2*-sensitive gradient echo sequence (resolution: 2.4 mm x 2.4 mm x 2.4 mm, TR: 1000 ms, TE: 30 ms, FoV: 192 mm, flip angle: 62°, multiplexing factor of 3).<br><br>Diffusion MRI: Data was acquired using a dual-spin echo sequence in 96 different directions, 8 non-diffusion-weighted (b=0) images were collected, 60 slices provided full head coverage (resolution: 2 mm × 2 mm × 2 mm, TR: 8000 ms, TE: 93.6 ms, FoV: 220 mm, flip angle: 90°, b: 2000 s mm-2).<br><br>Quantitative MRI: T1 relaxation times were measured from four spoiled gradient echo images with flip angles of 4°, 10°, 20° and 30° (TR: 14 ms, TE: 2.4 ms). The resolution of these images was later resampled from 0.8x0.8x1.0 mm^3 to 1mm^3 isotropic voxels. We also collected four additional spin echo inversion recovery (SEIR) scans with an echo planar imaging read-out, a slab inversion pulse and spectral spatial fat suppression (TR: 3 s, resolution: 2 mm x 2 mm x 4 mm, echo time set to minimum full, 2x acceleration, inversion times: 50, 400, 1200, and 2400 ms). The purpose of these SEIRs was to remove field inhomogeneities.
Area of acquisition	whole-brain in all data types

Diffusion MRI ☒ Used ☐ Not used

Parameters: Diffusion-weighted MRI (dMRI) data was collected in the same participants, at the same facility and with the same 32-channel head-coil as other data. DMRI was acquired using a dual-spin echo sequence in 96 different directions, 8 non-diffusion-weighted (b=0) images were collected, 60 slices providing full head coverage, TE=93.6ms, TR=8000ms, b=2000s/mm2, voxel size=2×2×2mm.

Preprocessing

Preprocessing software	Anatomical MRI was segmented using FreeSurfer; Manual corrections of the FreeSurfer segmentation were done in ITKgray.<br><br>Functional MRI was motion corrected, aligned to each subject's anatomical MRI, high pass filtered to remove drift and transformed to percent signal change in Vistasoft. No spatial smoothing was applied.<br><br>Diffusion data was preprocessed using Vistasoft and Mrtrix3: data was motion corrected, eddy current corrected, and aligned to the subject's anatomical MRI.
Normalization	No normalization; All data are analyzed in the native brain space of each participant.
Normalization template	N/A
Noise and artifact removal	functional MRI: Both within and between scan motion correction, as well as, high pass filtering to remove fMRI drift.<br>diffusion MRI: Motion correction and eddy current correction.
Volume censoring	N/A

Statistical modeling & inference

Model type and settings	Univariate approach, we used a general linear model (GLM) in each voxel, and the output was used to define regions of interest for subsequent analyses.

Effect(s) tested

We defined three kinds of task related regions using functional MRI:
Voxels which showed significantly higher responses during reading than adding or color judgments (t>3, voxel level)
Voxels which showed significantly higher responses during adding than reading or color judgments (t>3, voxel level)
Voxels which showed significantly higher responses during color than adding or reading judgments (t>3, voxel level)
In addition we defined conjunction voxels that responded more to adding than color task and more to reading than color task

Specify type of analysis: ☐ Whole brain ☐ ROI-based ☒ Both

Anatomical location(s)

Anatomical locations were determined in each subject's brain based on the identification of specific macroanatomical landmarks (sulci and gyri)

Statistic type for inference
(See Eklund et al. 2016)

voxel-wise, t≥3

Correction

none

Models & analysis

n/a	Involved in the study
☒	☐ Functional and/or effective connectivity
☒	☐ Graph analysis
☐	☒ Multivariate modeling or predictive analysis

Multivariate modeling and predictive analysis

We implemented a support vector machine (SVM) to classify tracts into 2 classes: those that are involved in mathematical processing and those that are involved in reading. Training set: nodes of fascicles; Testing set: the fifth more posterior node relative to the training nodes.
